# A two years longitudinal study of a transgenic Huntington disease monkey

**DOI:** 10.1186/1471-2202-15-36

**Published:** 2014-03-03

**Authors:** Anthony WS Chan, Yan Xu, Jie Jiang, Tayeb Rahim, Dongming Zhao, Jannet Kocerha, Tim Chi, Sean Moran, Heidi Engelhardt, Katherine Larkin, Adam Neumann, Haiying Cheng, Chunxia Li, Katie Nelson, Heather Banta, Stuart M Zola, Francois Villinger, Jinjing Yang, Claudia M Testa, Hui Mao, Xiaodong Zhang, Jocelyne Bachevalier

**Affiliations:** 1 Division of Neuropharmacology and Neurologic Diseases, Yerkes National Primate Research Center, Emory University, Atlanta, Georgia; 2 Department of Human Genetics, Emory University School of Medicine, Atlanta, Georgia; 3 Yerkes Imaging Center, Yerkes National Primate Research Center, Emory University, Atlanta, Georgia; 4 Department of Psychiatry and Behavioral Sciences, Emory University School of Medicine, Atlanta, Georgia; 5 Department of Pathology and Laboratory Medicine, Emory University School of Medicine, Atlanta, Georgia; 6 Division of Pathology, Yerkes National Primate Center, Emory University, Atlanta, Georgia; 7 Department of Neurology, Emory University School of Medicine, Atlanta, Georgia; 8 Department of Radiology and Imaging Sciences, Emory University School of Medicine, Atlanta, Georgia; 9 Department of Psychology, Emory University School of Medicine, Atlanta, Georgia; 10 Division of Developmental and Cognitive Neuroscience, Yerkes National Primate Center, Emory University, Atlanta, Georgia

## Abstract

**Background:**

A two-year longitudinal study composed of morphometric MRI measures and cognitive behavioral evaluation was performed on a transgenic Huntington’s disease (HD) monkey. rHD1, a transgenic HD monkey expressing exon 1 of the human gene encoding huntingtin (*HTT*) with 29 CAG repeats regulated by a human polyubiquitin C promoter was used together with four age-matched wild-type control monkeys. This is the first study on a primate model of human HD based on longitudinal clinical measurements.

**Results:**

Changes in striatal and hippocampal volumes in rHD1 were observed with progressive impairment in motor functions and cognitive decline, including deficits in learning stimulus-reward associations, recognition memory and spatial memory. The results demonstrate a progressive cognitive decline and morphometric changes in the striatum and hippocampus in a transgenic HD monkey.

**Conclusions:**

This is the first study on a primate model of human HD based on longitudinal clinical measurements. While this study is based a single HD monkey, an ongoing longitudinal study with additional HD monkeys will be important for the confirmation of our findings. A nonhuman primate model of HD could complement other animal models of HD to better understand the pathogenesis of HD and future development of diagnostics and therapeutics through longitudinal assessment.

## Background

Huntington’s disease (HD) is an autosomal dominant neurodegenerative disorder caused by the expansion of a CAG triplet repeat in exon 1 of the *huntingtin* (*HTT*) gene, translated into a polyglutamine tract in the HTT protein. HD is a devastating disorder with progressive decline in motor, cognitive and psychiatric functions. Motor impairment, such as chorea, is one of the earliest clinical signs for diagnosis, whereas cognitive decline and psychiatric disturbances often precede the onset of motor dysfunction [1-11]. Although individual mutation status can be determined by genetic testing, currently available treatments are limited to symptomatic management to improve the patients’ well-being. Treatment does not alter or arrest the progressive development of dementia, bradykinesia, incoordination and rigidity that leads to disability [9,12,13].

In order to identify targets for developing and validating novel therapeutic approaches and new drugs for genetic and biochemical intervention, animal models including insects, rodents and large animal models such as ovine, pig and monkey have been developed [14-20]. Although these model systems have been useful in explaining HD pathogenesis and the development of potential treatments, limitations in longitudinal measurements (e.g. morphometry study of brain structures and cognitive behavioral assessments similar to those used in human patients) are major limitations for advancing clinical applications. Unlike other available animal models, transgenic HD monkeys develop dystonia and bradykinesia similar to HD patients when under stress and can be used for clinical evaluation procedures performed on HD patients [19]. The development of motor impairment reported in HD monkeys suggests a similarity in motor deficits between non-human primates and human HD [19], however, longitudinal changes in morphometric brain MRI measurement and cognitive functions in HD monkeys have not been reported. This report describes a two-year longitudinal study using morphometric measurement of brain structures obtained from non-invasive magnetic resonance imaging (MRI) and cognitive behavioral assessments in one of the first transgenic HD monkeys, rHD1 [19].

## Methods

### HD and control monkeys

A male transgenic HD monkey, rHD1, was created by transfection of mature oocyte by using lentivirus carrying a mutant *HTT* gene composed of Exon 1 of the *HTT* gene with extended CAG tract under the regulation of a human polyubiquitin C promoter as described previously [19]. rHD1 carries a single copy of the mutant *HTT* transgene with 29 CAG repeats. rHD1 and four age-matched wild-type control rhesus macaques (Males: REm12 and RWl12; Females: RCk12 and RFk12) were raised in the primate nursery under the same conditions in the same room. They all received the same treatments and procedures designed for the longitudinal study including MRI scans at every six months and cognitive behavioral studies. Physical measurements including body weight and head circumference were taken monthly, but only data at approximately six months intervals was presented.

### Quantitative measurement of mHTT transcript in peripheral blood

Total RNA isolation from the peripheral blood cells were collected from the HD monkey and controls. In brief, peripheral blood cell was homogenized in 500 μl of Trizol (Invitrogen). A phenol-chloroform extraction of the RNA was done by addition of 100 μl of chloroform to the Trizol homogenates followed by centrifugation at 12,000 × g for 10 minutes at 4°C. The aqueous layer was removed for RNA precipitation overnight with isopropanol at -20°C. The precipitated RNA was pelleted at 12,000 × g for 30 minutes at 4°C. All RNA pellets were washed twice with 75% ethanol and then subsequently dissolved in water (RNase/DNase free). 750 ng of total RNA was reverse transcribed to cDNA with the High Capacity Reverse Transcription Kit (Applied Biosystems). Quantitation of *HTT* transcript levels were evaluated by qPCR using a custom-designed gene-specific Taqman assay (Applied Biosystems) and reactions run on the BioRad CFX96 cycler. HTTexon1 Taqman assay (Forward primer: GCCGCTGCTGCCTCA; Reverse primer: TGCAGCGGCTCCTCAG; and Probe: CCGCCGCCCCCGCC). To determine relative expression level of mHTT transgene, Taqman assay for HTTexon26 was used (Forward primer: GCAGCCACCAAGCAAGAG; Reverse primer: GAGAAGAGCTGCTCCACCAT; and Probe: CAAGGCCCGGTCCCC). All data were normalized with the geometric mean of GAPDH and β-actin with Taqman assays.

### Western blot analysis

Total proteins were extracted from different tissues and their concentration was determined by Bradford assay (Pierce, Inc.). Equal amounts (20–30 μg) of protein extract with loading dye were boiled prior to loading into 4-15% gradient polyacrylamide gel (Bio-Rad, Inc.). Following electrophoresis, proteins were transferred onto a PVDF membrane (Bio-Rad, Inc.) using Bio-Rad’s transblot followed by blocking in 5% skim milk for 2 hours. The membrane was then incubated with primary antibodies, mouse monoclonal mEM48 (1:50 dilution)^(19)^, and γ-tubulin (Sigma; 1:2000 dilution), followed by secondary antibody conjugated with peroxidase (Jackson ImmunoResearch laboratories, Inc) for detecting proteins with an Amersham ECL kit (PerkinElmer, Inc.).

### Rearing conditions

After delivery, they were surrogate-nursery reared in the primate nursery of the Yerkes National Primate Research Center (YNPRC; Atlanta, GA) according to procedures developed by Sackett and colleagues [21] that allow normal growth as well as the development of species-specific social skills. These procedures included daily social interactions with peers, intensive human contacts, and cognitive testing that began in the first weeks of life and continued through adulthood (for additional details on rearing conditions, see Goursaud and Bachevalier, [22]).

Their diet consisted of infant Similac formula (SMA with iron) supplemented with banana pellets starting at 3–4 weeks old (190 mg, P.J. Noyes, Cleveland, OH). Starting around 8 months of age, they were fed jumbo primate chow (Lab Diet #5037, PMI Nutrition International Inc., Brentwood, MO) and fresh fruit daily. All experimental procedures were approved by the *Institutional Animal Care and Use Committee* of Emory University (Atlanta, GA) and were conformed to the NIH guide for the *Care and Use of Laboratory Animals*.

### MRI measurements

Longitudinal morphometric measurements using three-dimensional T_1_-weighted MR images were acquired with a Siemens 3T Trio whole body scanner (Siemens Medical, PA, USA) with the Siemens CP extremity volume coil on all animals. Scans were performed every six months for two years, starting at six months of age. Animals were immobilized with a custom-made head holder and placed in the sphinx position. Anesthesia was maintained with 1–1.5% isoflurane mixed with O2. Et-CO_2_, inhaled CO_2_, O_2_ saturation, blood pressure, heart rate, respiration rate, and body temperature were monitored continuously. Three-dimensional (3D) T1-weighted images with isotropic resolution were acquired by using the MP-RAGE sequence with the parameters: TR = 2500 ms, TE = 3.48 ms, TI = 950 ms, FOV = 96 mm × 96 mm, data matrix = 192 × 192, flip-angle = 8 degree, slice thickness = 0.5 mm, 208 slices, 4 averages.

### Brain volume calculation

The 3D T1-weighted images were used for the total and regional brain volume calculation. The skull was stripped from all images before the calculation of total brain volume (TBV). To perform brain extraction, we use the FSL Brain Extraction Tool (BET; http://www.fmrib.ox.ac.uk/fsl) [23,24]. Hippocampal and striatal (caudate and putamen) volumes were measured based on anatomy delineated on coronal T1-weighted images across the two structures and manually traced. Regional and TBV volumes were then calculated by using Image 1.42q software. For comparison, regional volume was normalized with the TBV.

### Huntington’s disease primate model rating scale (HDPMRS) [19]

HDPMRS was modified from the Unified Huntington’s Disease Rating Scale (UHDRS) [25], which is commonly used for monitoring progression of HD in patients, and to evaluate the progression of motor and cognitive functions that classically accompany the disease. The HDPMRS is primarily focused on monitoring the progression of motor impairment while psychological behaviors, such as suicidal thought and speech were excluded. The first section of HDPMRS was focused on motor ability and the second section was focused on functional assessment (see Additional file 1: Table S1 for details on HDPMRS). Video recording and scoring was performed every 12 months for 30 minutes in a cage inside the room where the animals were housed.

### Behavioral testing

All animals were tested at different points during development to follow their neurobehavioral and motor development and to assess functioning of different cognitive systems. Additional file 1: Table S2 displays the list of behavioral tasks given to each animal and the age at which each task was administered. All behavioral tasks were conducted in a sound-attenuated room equipped with a white noise generator to reduce external noise, except otherwise mentioned. For the 1-Pair Object Discrimination (1-pair OD), Pattern Discrimination (PD), 24-hr Concurrent Object Discrimination (COD), Object Discrimination Reversal (ODR), Delayed Nonmatching-to-Sample (*DNMS*), Detour-reaching/barrier and Visuopatial Orientation (VS-OR), animals were transferred to a Wisconsin General Testing Apparatus (WGTA), facing a test tray onto which objects or equipment could be positioned. For the Visual-Paired Comparison (VPC) tasks, animals were placed within an enclosure facing a TV monitor onto which pictures could be displayed. Rewards for problem solving tasks were either peanut, raisin, fruity gems, mini M&M, or Marshmallows, depending on animal’s preference.

### Infant neurobehavioral assessment scale (INAS)

The INAS is an assessment instrument developed by Schneider and Suomi (1992) [26] to measure maturation of a wide range of neonatal behaviors (orientation behavior, neuromotor abilities, and temperament measures) in newborn monkeys. This instrument was administered for 20 minutes at the following postpartum days: 5–9, 13–18, 20–25, and 27–35 days between 10:00AM and 12:00PM in the primate nursery with dimmed lights. Three categories of behavior were tested. First, orienting responses were elicited by a plastic toy (visual orienting) and a noise (auditory orienting) while the infant was wrapped in a towel and hand-held by an experimenter. Next, neuromotor functions were assessed with the infant placed in a warm flat surface and included muscle tonus, coordination, balance, response speed and spontaneous motor activity. Finally, temperament ratings were assessed during the orienting and neuromotor procedures and included vocalizations, fearfulness, irritability, and consolability. Additional file 1: Table S3 provides a description of the behavioral measures and of the rating scale used for each of the three categories.

### Measures of stimulus-reward associations

To assess functioning of the striato-cortical loop, we used behavioral tasks for which performance is known to be affected by lesions of the striatum [27-29]. In addition, given that we needed to measure striatal-dependent functions across development, stimulus-reward association learning tasks used at each age increased in difficulty from the simplest 1-pair OD given at 4 and 8 months to the more challenging pattern discrimination (PD) given at 8 months and 20-pair concurrent discrimination (COD*)* at 9 months of age. (Please see Additional file 2 for detailed procedures).

### Measure of flexible behavioral and cognitive inhibition and impulsivity

To assess the functioning of the frontal cortex, we use two sensitive tasks: object discrimination reversal (ODR) and Detour-reaching/barrier task at 12 months and 16 months, respectively. (Please see Additional file 2 for detailed procedures and Additional file 1: Table S4).

### Measures of object and spatial recognition memory functions

Sensitive measures of medial temporal lobe and hippocampal functions were assessed with two recognition memory tasks: VPC tasks (1, 4, and 8 months) and DNMS (16 months). (Please see Additional file 2 for detailed procedures).

### Measures of visuospatial abilities

To assess the contributions of the frontal-striatal system to fine motor control and associative learning, we use VS-OR (or lifesaver task) at 16 months of age. (Please see Additional file 2 for detailed procedures).

## Results

### Expression of mutant HTT in peripheral blood and lymphoblast cell lines

**Figure 1:**
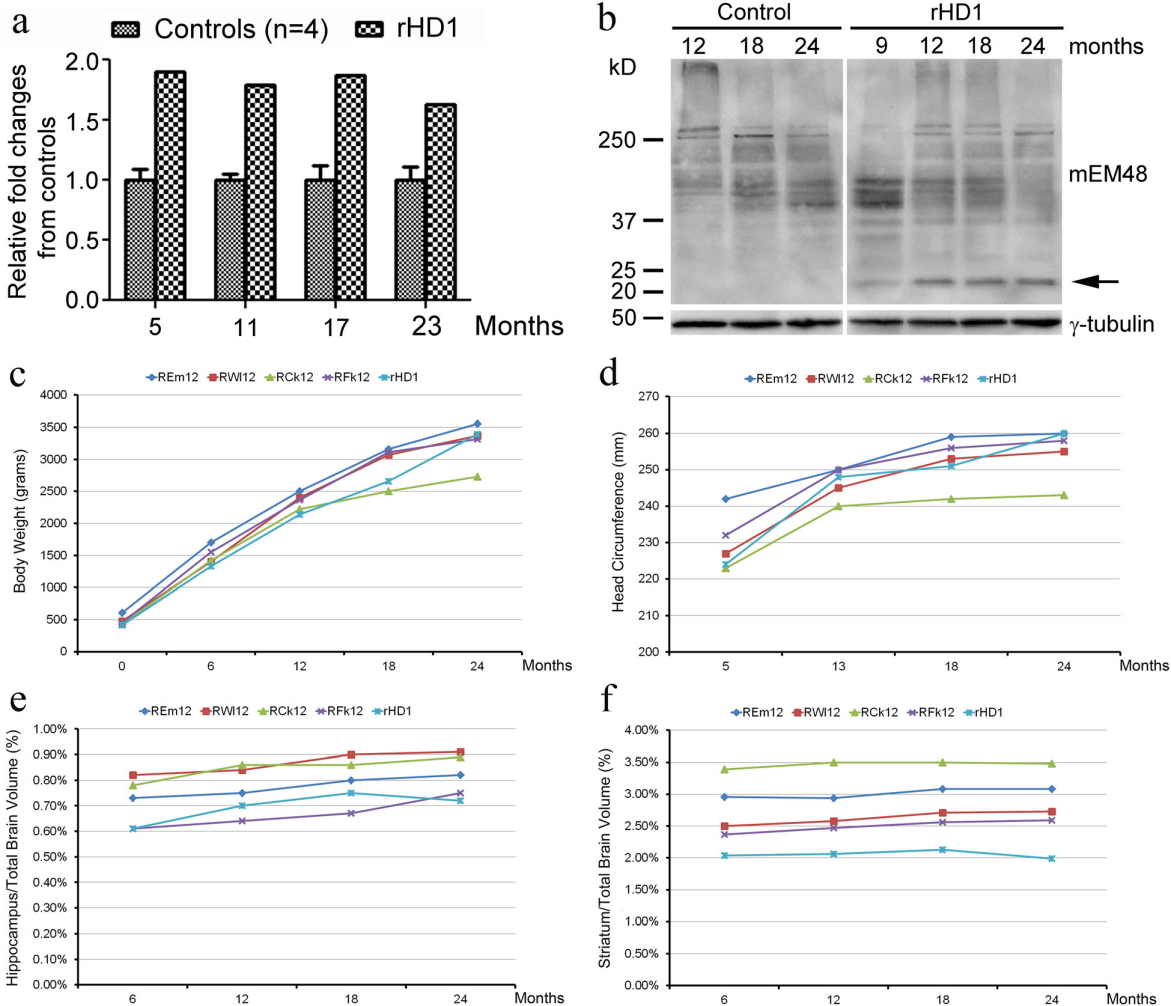
**Expression pattern of mutant HTT and brain volumetric measurements in a transgenic HD monkey and control monkeys. (a)** Quantitative measurement of mutant *HTT* transcript in controls and rHD1 peripheral blood at four time-points by quantitative PCR, **(b)** Western blot analysis of one of the control monkeys and rHD1 at various time points. Arrow indicates the expression of soluble form mutant HTT protein that was not observed in control monkey samples. **(c)** Longitudinal measurement of body weight. **(d)** Longitudinal measurement of head circumference. Longitudinal morphometric measurements of **(e)** hippocampus and **(f)** striatum at four time-points by MRI normalized with total brain volume.

rHD1 carries a single copy of the mutant *HTT* gene with human exon1 and 29 CAGs regulated by the polyubiquitin C promoter [19]. Wild-type rhesus macaques carry an average of 10–11 CAGs [30]. Expression of mutant *HTT* was measured in peripheral blood cells by Q-PCR (Figure 1a). Mutant *HTT* transcript was approximately one fold higher than the endogenous *HTT* level in comparison to the control monkeys while it remained at similar levels as the disease progressed over the two-year period. Soluble form mutant HTT protein was detected in lymphoblast cell lines established at four time points (9, 12, 18 and 24 months) by western blot analysis, but robust oligomeric mutant HTT aggregates were not observed compared to control monkey lymphoblast cells (Figure 1b).

### HD primate model rating scale (HDPMRS; Additional file 1: Table S1)

rHD1 has a total score of 6 versus an average of 1 (0, 0, 2 and 2) for the controls at 24 months of age while a score of “0” was recorded for rHD1 and control monkeys at 12 months of age. rHD1 scores were mainly in lower limb dystonia. He also had several episodes of tonic-clonic seizure at around 22 months of age when under stress such as in transfer cage during cage wash.

### Physical measurements

rHD1 had growth trajectory in body weight (Figure 1c) and head size (Figure 1d) similar to the four wild type controls through the first two years of age. Among the control monkeys, only one female (RCk12) had a lower body weight and smaller head size compared to rHD1.

### Morphometric measurement of striatal and hippocampal volumes

When controlled for TBV, the hippocampal volume in rHD1 showed a similar growth pattern as the controls, but was smaller as compared to controls, except for one female control (RFk12) (Figure 1e). The slight reduction of hippocampal volume in rHD1 became more pronounced at 24 months of age (Figure 1e). Striatal volume of rHD1 showed a similar developmental trajectory as the controls (Figure 1f) with an overall smaller striatal size across all ages.

### Infant Neurobehavioral Assessment Scale (INAS) [26]

**Figure 2:**
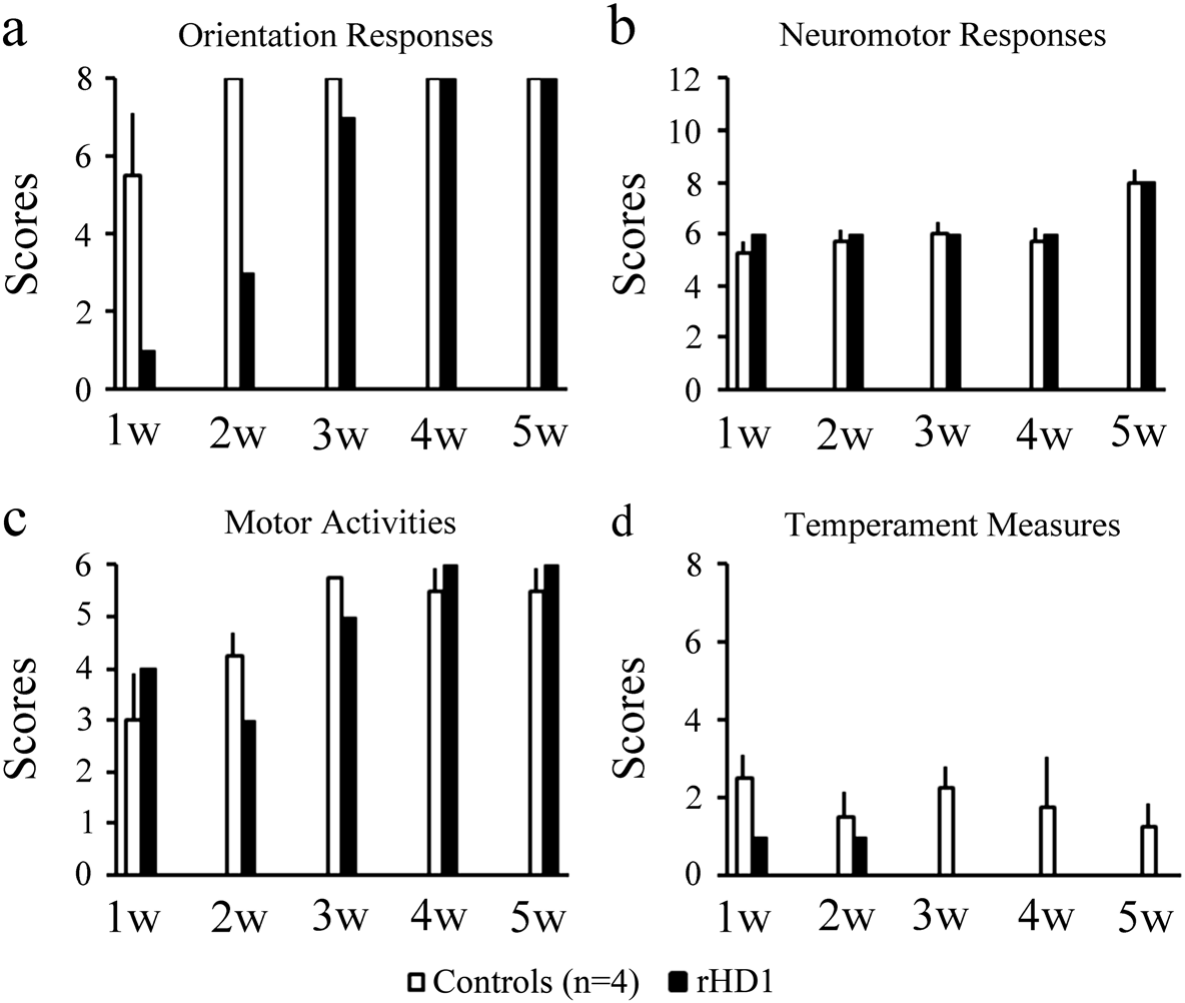
**Infant neurobehavioral assessment scale results.** Scores are rated for **(a)** orienting responses (visual orienting and following, attention span, auditory orienting), **(b)** neuromotor abilities (muscle tonus prone and supine, pull-to-sit, coordination and response speed), **(c)** motor activities (locomotion and coordination), and **(d)** temperament (vocal reactions, fearfulness, struggle, irritability, consolability during testing) for four controls (white bar) and rHD1. Controls are represented by mean value with SEM error bar.

Figure 2 illustrates scores of rHD1 and scores of the four control animals for orientation responses, neuromotor responses, motor activities, and temperament on the INAS scale during the first five weeks of life. rHD1 had scores comparable to those of controls for all measures, except orientation responses. rHD1 demonstrated weaker visual and auditory orienting responses during the first two weeks after birth compared to controls. However, his orientation responses returned to control levels by weeks three to five.

### Measures of stimulus-reward associations

**Figure 3:**
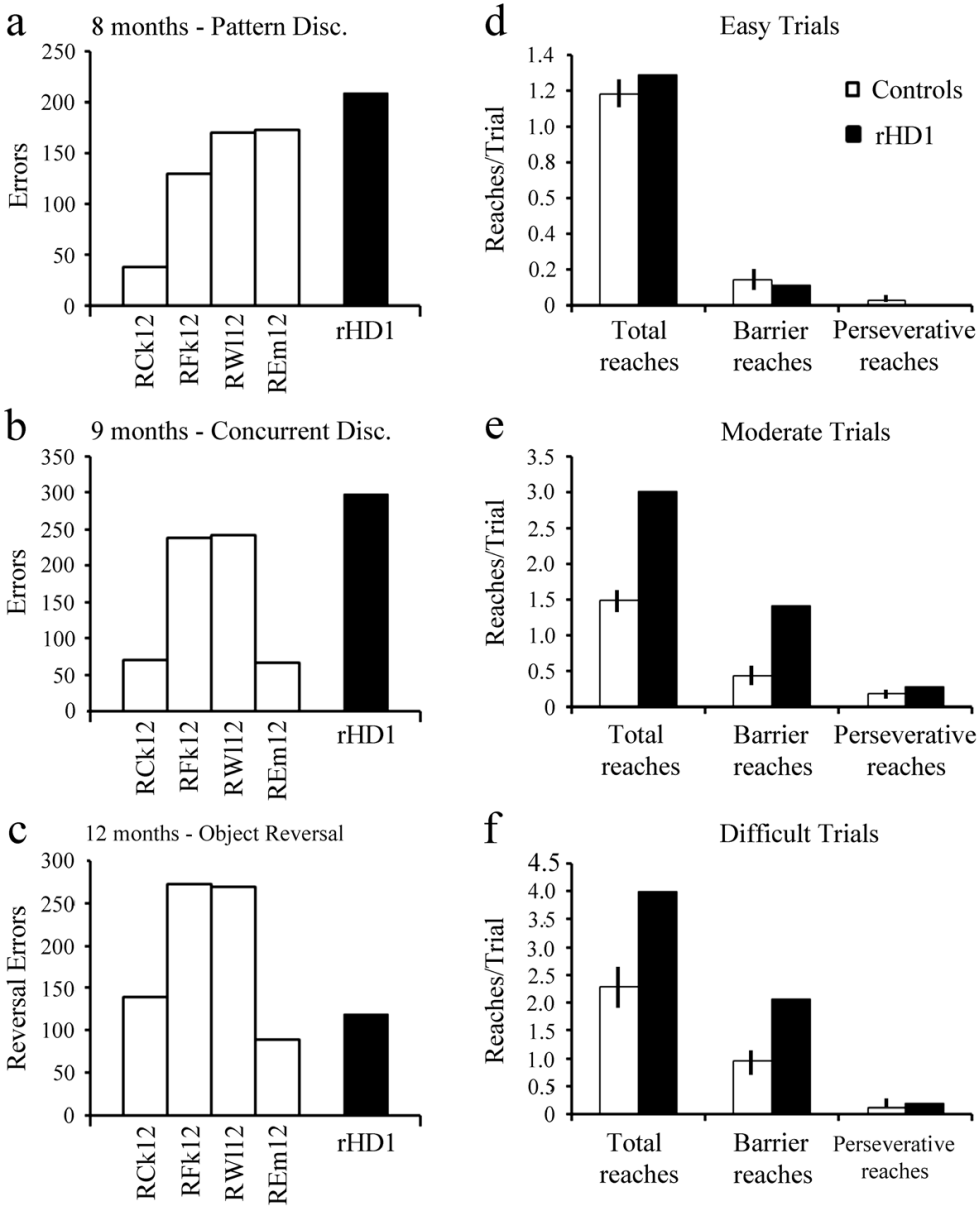
**For measures of stimulus-reward associations and flexible behavioral inhibition, scores are the number of errors committed until reaching the criterion for (a) pattern discrimination at eight months, (b) concurrent discrimination at nine months and (c) object discrimination reversal at 12 months for the four controls and rHD1.** For measures of cognitive inhibition and impulsivity, scores are number of total reaches, barrier reaches and perseverative reaches during the **(d)** “Easy” trials (box opened left or right and reward positioned at the box entrance), **(e)** “Moderate” trials (box opened right or left and reward positioned mid-way inside the box), and **(f)** “Difficult” trials (box opened right or left and reward positioned on the opposite side of the box) for the four controls (Mean ± SEM, open bars) and rHD1 (solid bars).

To reach criterion in the one-pair object discrimination at four months, rHD1 made 50 errors, which is similar to control animals (zero, seven, 24, and 106 errors). Again, when retested on a new discrimination problem at eight months of age, rHD1 performed even better than controls, relearning the task immediately (zero errors) as compared to the four control animals that averaged 60 errors (SEM: 30.0). Thus, rHD1 acquired simple object discrimination problems at the same rate as controls at both four and eight months (data not shown). For acquisition of a pattern discrimination problem at eight months, rHD1 was slightly delayed as compared to the four control animals, making 208 errors to reach criterion as compared to 38 to 173 errors for the controls (Figure 3a). At nine months of age, rHD1 failed to reach criterion of the 20 concurrent discrimination problems in the limit of testing, whereas all four controls reach criterion. The impairment in rHD1 was reflected by the increased number of errors committed (300 errors, Figure 3b) as compared to the four controls (66 to 242 errors).

### Measure of flexible behavioral and cognitive inhibition and impulsivity

Both object discrimination reversal task and detour-reaching/barrier task are sensitive tasks to measure functioning of the prefrontal cortex. At 12 months of age, all animals, including rHD1, learned the initial object discrimination reversal problems rapidly (0, 3, 16, 16 errors for the controls and 4 errors for rHD1). During the six reversals, again rHD1 performed as well as controls, totaling 398 reversal errors and 119 perseverative errors as compared to an average of 548 (range: 300–750) reversal errors and 193 (range: 90–272) perseverative errors for the controls (Figure 3c). On the other hand, at 16 months of ag*e,* rHD1 performed as well as the controls on the “easy” trials (Figure 3d) of the detour/barrier task, but this animal made almost twice as many barriers and perseverative reaches in the “moderate” (Figure 3e) and “difficult” (Figure 3f) trials than did control animals.

### Measures of object and spatial recognition memory functions

**Figure 4:**
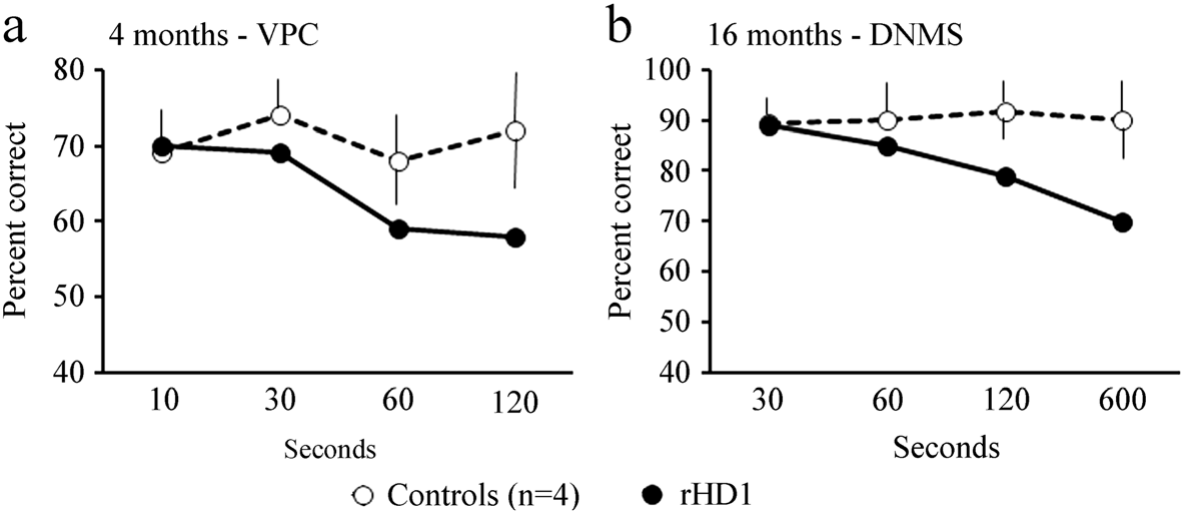
**The impairment in hippocampal-dependent memory functions in rHD1.** Scores are percent correct when looking at novel objects at four different delays **(a)** 10s, 30s, 60s, 120s in the VPC (*Visual Paired Comparison)* task at four months and **(b)** percent correct at delays of 30s, 60s, 120s, and 600s in the DNMS (Delayed Nonmatching-to-Sample) task at 18 months for the four controls (open circle and dashed line) and rHD1 (close circles and solid line). Controls are represented by mean value with SEM error bar.

Sensitive measures of medial temporal lobe and hippocampal functions were assessed with two recognition memory tasks, VPC and DNMS. At one month of age, rHD1 showed strong novelty preference and obtained scores averaging 62%, 63%, 60% and 59% at 10s, 30s, 60s and 120s delays in VPC respectively, only slightly below those of control animals (69%, 67%, 63% and 69%). By contrast, at four months of age, although rHD1 continued to show novelty preference scores above chance, its scores at the longest delays of 60s and 120s were lower than those of controls (Figure 4a). The same impairment in recognition memory was also found in rHD1 as compared to the four controls, when all animals were re-tested at 16 months of age.At 16 months of age, as compared to controls that learned the DNMS task in an average of 310 trials and 71 errors, rHD1 was impaired, requiring 760 trials with 181 errors. Interestingly, in the performance test with increasing delays, rHD1 performed as well as controls at short delays of 30 and 60s, but his performance declined below that of controls as the delays further increased to 120 and 600s (Figure 4b).

As shown in Table 1, in the spatial location VPC, rHD1 obtained scores above chance levels, although his novelty preference was weaker (59%) than that of the four controls (range: 68%-75%). rHD1 was even more severely impaired on the object-in-place VPC compared to controls. In this relational spatial recognition task, rHD1’s novelty scores were at chance levels (48%) and lower than those of controls (range: 56%-66%). This impairment cannot simply have resulted from the poor visual search of all objects on the pictures, since rHD1 showed strong recognition memory in the object-control task (61%), which also required looking at pictures consisting of five objects.

**Table 1:** **Percent looking at novel in the spatial *Visual Paired Comparison (*** **VPC) tasks**

	**Spatial-location**	**Object-in-place**	**Object-control**
REm12	71.1	55.9	62.6
RWl12	68.8	65.8	77.6
RCk12	68.3	55.9	76.7
RFk12	75.5	58.0	63.5
**rHD1**	**58.9**	**47.8**	**60.6**

### Measures of visuospatial abilities

As shown in Table 2, as compared to controls, rHD1 appeared to show some visuospatial difficulties with the “Easy” Routes, requiring longer time (28s) to thread the Lifesaver out of the rods and failing to free the candy in many more trials (18 failures) than any of the four controls (Range: 19-24s and 4–12 failures, respectively). However, this difference was absent in the “Difficult Routes”, suggesting that, with further practice, rHD1 visuospatial abilities returned to the levels of those of control animals.

**Table 2:** Visuospatial-orientation task

	**Mean time (sec)**	**Fastest time (sec)**	**Failures**
**Easy routes**			
REm12	19.71	1.87	5
RWl12	20.38	0.88	11
RCk12	19.37	2.13	4
RFk12	24.57	0.71	12
**rHD1**	**28.61**	**3.83**	**18**
**Difficult routes**			
REm12	25.67	4.25	1
RWl12	53.47	1.75	11
RCk12	31.34	4.00	3
RFk12	68.39	1.03	12
**rHD1**	**49.17**	**4.70**	**4**

## Discussions

We followed the progression of symptoms and brain maturation in the first transgenic HD monkey by monitoring longitudinal clinical measurements of motor functions, cognitive assessments involving several brain networks, and morphometric measurements of brain structures over a period of two years. rHD1 has similar growth trajectory compared to the control monkeys based on body weight gain (Figure 1c) and head size (Figure 1d). While weight loss occurs in HD patients despite adequate or even increased caloric intake [31,32], we expect weight loss in HD monkey may be revealed as disease progresses. A recent study of children at risk of HD showed a significant correlation between expanded polyQ and lower measurement of head circumferences, weight and body mass index [33], which differed from our observation. However, the growth trajectory of preHD children was not different from the controls [33]. No children with JHD were included in the study, which may account for the difference with rHD1 that had clinical development similar to human JHD. Although similar growth trajectory was observed between HD and control monkeys, the current study is based on the observation of a single HD monkey and more conclusive information should be expected from our ongoing longitudinal study of a group of HD monkeys with extended age. Nevertheless, the data demonstrate for the first time the course of pathological changes and clinical progression in the primate model of HD.

Although cognitive behavioral assessments are important clinical measurement on the progression of HD, and standardized tests are used, sensitive methods for quantitative measurement of cognitive behavioral functions are limited due to variations among patients and experimental testers as well as assessment batteries [1,5,7,8,25,34-36]. Discrepancy due to overestimation on the sensitivity of measurements in cross-sectional studies suggests the importance of unbiased longitudinal studies for precise interpretation of the results and for determining possible clinical applications [1,6-8,37,38]. Unlike the cognitive behavioral assessment, non-invasive MRI provides an optimal quantitative tool for determining anatomical changes that may associate with the disease progression. Several recent studies have shown specific regional atrophy in the brain, particularly in the caudate and putamen of presymptomatic HD mutation positive individuals or HD prodrome. MRI changes occur before functional and motor impairments, and that progression in MRI changes is correlated with development and progression of functional and motor impairments [1,7,8,37,39-43]. The hippocampus, which is critical for spatial and relational memory, is also reduced in pre-manifest HD and early HD [37,44-46]. Recent longitudinal studies on pre-manifest and early stage HD patients demonstrate significant progressive atrophy in the caudate, putamen, thalamus and nucleus accumbens [7,8,37,39,40,43,47,48]. The progression in brain atrophy paralleled the decline in cognition and motor functions in early HD [7,8,35]. These studies suggest that quantitative longitudinal neuroimaging is a powerful tool for differentiating pre-manifest and early stage HD and can be correlated with functional and motor measurements for monitoring disease status and progression rate [7,8,37,39,40,43,47,48].

A similar pattern of disease progression was observed in morphometric measurement of the striatal and hippocampal regions and in the decline of cognitive functions associated with these two brain regions in rHD1. The overall volume of both hippocampus and striatum was smaller in rHD1 at all ages. However, the head size of rHD1 was smaller at 5 months of age but continued to grow and was comparable to three of the four control monkeys up to 2 years of age except for one female control (RCk12) with a smaller head size. The cognitive decline was in fact present earlier than volumetric changes in the striatum and hippocampus, when no obvious motor deficits were yet noticed. Indeed, rHD1 had a HDPMRS score of six versus an average of one for the controls at 24 months. rHD1 scores were mainly in dystonia, which occurred during cognitive testing when he was transferred into a testing apparatus at a different location. An episode of seizure occurred at 22 months of age during the time of cage cleaning. In general, motor dysfunction and seizure often occurred when rHD1 was under stressful conditions. Cognitive changes were first clear at eight months of age; in this species, 48 months is considered adulthood. Developmental trajectory between rhesus macaques and humans is an estimation of 1 year old monkey *vs* 4 years old humans, respectively. Given the age of rHD1, the imaging findings and progressive development of cognitive behavioral decline, especially seizures, this particular animal may parallel human juvenile onset HD (JHD) even only with a small expansion of polyglutamine (29Q) compared to normal rhesus macaque (10-11Q) at the *HTT* gene. JHD patients have motor symptom onset younger than age 21, with a higher incidence of seizures in very young onset cases and stiffness, parkinsonism, and dystonia outweighing chorea especially in very young onset cases [49-54]. Seizures are unusual in adult onset HD [49]. Imaging data in human JHD is very sparse, with caudate atrophy being the most consistent finding [55-58], which parallels with our findings that reduction in caudate volume was more profound compared to the putamen. rHD1 does exhibit chorea, but also exhibits early cognitive decline and seizures.

rHD1 showed deficits in pattern discrimination and concurrent discrimination learning that were present and detectable as early as eight and nine months of age (Figures 3a, b), suggesting striatal dysfunction that preceded the striatal atrophy detectable by 24 months of age (Figure 1f). This impairment was reminiscent of the impairment in pattern recognition memory found in HD patients in early stages of the disease, which has been associated with ventrocaudal striatum dysfunction [45]. Longitudinal studies over the full range of clinical manifestation stages are essential for determining the progression of disease, to identify biomarkers, and to develop novel therapeutic targets. There are longitudinal studies in humans, some ongoing, allowing analysis of face and construct validity in the HD transgenic primate model. A recent report of a 12-and-46 month longitudinal analysis indicates caudate atrophy in pre-manifested HD patients [7,8] compared to the control group, although similar findings have also been reported in JHD patients [55-58]. A similar result was also observed in our transgenic HD monkey.

Dysfunction of the hippocampus in rHD1 became detectable as early as four months of age and was further demonstrated at 16 months by deficits in the delayed non-matching task and in both VPC tasks (Figure 4), respectively. The impairment in hippocampal-dependent memory functions was consistent with the reduction in hippocampal size at 24 months of age (Figure 1e). These findings replicate the hippocampal-dependent memory deficits found in HD patients even before the onset of motor symptoms [5,7,8,45,59,60], which have also been reported in all analyzed mouse models of the disease [11,20,44,61].

Frontal cortical deficits present in HD patients [62,63] were also apparent in rHD1 at 16 months of age, as shown by increased cognitive impulsivity (increased barrier and perseverative reaches) in the Detour-Reaching task (Figure 3 Right panel), indicating dysfunction of fronto-striatal circuitry. At this same age, visuospatial abilities were only transiently altered in rHD1 as reflected by longer latency to free the treat and greater number of failures to retrieve the reward; however, this deficit was observed only when the animals were tested with the “Easy”, but not with the “Difficult”, routes. Although this pattern of results may indicate habituation to the task and practice effect, it is also possible that greater impairment in visuospatial function may emerge later during the progression of this animal’s disease state. Interestingly, decline in visuospatial abilities during the HD prodromal phase has not been consistently reported in the literature, but when present, it is often found in patients closer to disease onset (see for review Papp et al., [64]). Finally, rHD1 showed normal performance on the simple discrimination reversal task, consistent with the spared simple reversal learning abilities found in early HD patients [45].

## Conclusions

To summarize, our results demonstrate disease progression of a transgenic HD monkey based on longitudinal cognitive behavioral assessments and volumetric MRI measurements. The progression of rHD1 is closer to juvenile onset HD than adult onset with the development of seizures, early impact on cognition and mild motor impairment [49-54]. We acknowledged that additional animals need to be prepared and studied using the same behavioral and cognitive tasks as well as neuroimaging procedures across development. However, given the length of time it requires to produce this type of animals and to assess behavioral and brain changes across development, we believe that the longitudinal study on this first transgenic HD monkey is encouraging and important, and suggest their potential role in understanding HD pathogenesis, identifying biomarkers and as a preclinical animal model for developing new therapeutics.

## Competing interests

The authors declare that they have no competing interests.

## Authors’ contributions

AWSC planned, designed, oversaw all studies, data analysis and interpretation, wrote and approved the paper. YX performed HDPMRS and preparation of the manuscript. JJ, TR, HC and CL performed MRI analysis. JK performed expression analysis. DMZ performed western analysis. TC, HE, SM, KL, AN, HB and KN provided animal care and performed cognitive behavioral tests. SMZ consulted on cognitive behavioral study and edited paper drafts. FV the establishment of lymphoblast cell lines. JY the establishment of lymphoblast cell lines and technical support. CMT consulted on the HDPMRS and edited paper drafts. HM and XZ design and analysis of MRI study. JB planned and analyzed cognitive behavioral studies and wrote the paper. All authors read and approved the final manuscript.

## Supplementary Material

Additional file 1: Table S1 Huntington’s Disease Primate Motor Rating Score (HDPMRS). **Table S2.** Behavioral testing schedule. **Table S3.** INAS behaviors and rating scale. **Table S4.** Definitions of behavioral measures in the Detour-Reaching Task. Click here for file

Additional file 2 Supplemental Methods: Behavioral Testing. Click here for file
