# Supplementary material for: A two years longitudinal study of a transgenic Huntington disease monkey
Source: BMC Neurosci. 2014 Mar 3;15:36. doi: 10.1186/1471-2202-15-36 (PMC4015530; doi:10.1186/1471-2202-15-36)
Supplement: Additional file 1: Table S1 — Huntington’s Disease Primate Motor Rating Score (HDPMRS). Table S2. Behavioral testing schedule. Table S3. INAS behaviors and rating scale. Table S4. Definitions of behavioral measures in the Detour-Reaching Task. [file 1471-2202-15-36-S1.docx]

**Supplemental Table 1: Huntington’s Disease Primate Motor Rating Score (HDPMRS)**

|  | | | **Score** |
| --- | --- | --- | --- |
| Motor ability | | |  |
| Bradykinesia | | |  |
| Rigidity | Upper extremity | Left |  |
|  |  | Right |  |
|  | Upper extremity | Left |  |
|  |  | Right |  |
|  | Neck |  |  |
|  | Tail |  |  |
| Dystonia | Upper extremity | Left |  |
|  |  | Right |  |
|  | Upper extremity | Left |  |
|  |  | Right |  |
|  | Neck |  |  |
|  | Trunk |  |  |
| Chorea | Upper extremity | Left |  |
|  |  | Right |  |
|  | Upper extremity | Left |  |
|  |  | Right |  |
|  | Trunk |  |  |
|  | Face |  |  |
| Diagnosis confidence level |  |  |  |
| **Total score** | | |  |

**Motor assessment**

**Motor ability**: The quality and ability of locomotion is assessed. Scale is:

“0”=normal (walking and climbing without difficulty)

“1”=walking and limited ability to climb (only able to climb for a short time span/ limited height)

“2”= walking but not unable to climb

“3”=walking with difficulty (possess a weak ability to walk)

“4”=none (unable walk or stand)

**Bradykinesia**: means slow moving and impaired ability to adjust the body’s position. Scale is:

“0”=normal (movements occur normally, without hesitation)

“1”=minimally slow (movement is minimally slow, slightly slower than normal)

“2”= mild but clearly slow (movement is undoubtedly slower than normal)

“3”= moderately slow with some hesitation (movement is slow with slight hesitation, lasting less than a couple seconds)

“4”= markedly slow and long delays in hesitation (movement are clearly slow and the animal hesitates for more than 2 seconds)

**Rigidity**: the examiner rates the stiffness/tenseness of muscles in the arms, legs, neck and tail. Scale is:

“0”=absent (limbs can easily be bent and no stiffness is observed)

“1”=slight (muscles exhibit a slight amount of tension)

“2”=mild or moderate (muscles are moderately tense)

“3”=severe with full range of motion (muscles are extremely tense however they are still capable of full range of movement)

“4”= severe with limited range (muscles are extremely rigid and are unable to move with full range)

**Dystonia**: the frequency and severity of dystonia is assessed. Dystonia is described as disordered tonicity of muscles which results in prolonged involuntary muscle contractions leading to twisting body motions, tremors and abnormal posture. Dystonia can involve the entire body or an isolated area, thus the parts of the body are addressed independently. Scale is:

“0”=absent (nothing resembling dystonia is observed)

“1”=slight/intermittent (slight expression observed occasionally)

“2”=mild/common or moderate/intermittent (slight expression frequently or moderate expression observed occasionally)

“3”=moderate/common (moderate expression observed frequently)

“4”=marked/prolonged (definite expression observed continuously)

**Chorea:** the frequency and severity of chorea is assessed. Chorea is characterized by brief irregular contractions that are not repetitive or rhythmic but nevertheless appear to flow from one muscle to the next. Chorea is also rated in individual areas of the body. Scale is:

“0”=absent (nothing resembling chorea is observed)

“1”=slight/intermittent (slight expression observed occasionally)

“2”=mild/common or moderate/intermittent (slight expression frequently or moderate expression observed occasionally)

“3”=moderate/common (moderate expression observed frequently)

“4”=marked/prolonged (definite expression observed continuously)

**Diagnosis confidence level**: The examiner rates their confidence in which the subject’s motor symptoms, if any, are related to the onset and presence of HD. Scale is:

“0”=normal (no abnormalities)

“1”=non-specific motor abnormalities (less than 50% confidence)

“2”=motor abnormalities that may be signs of HD (50-89% confidence)

“3”=motor abnormalities that are likely signs of HD (90-98% confidence)

“4”=motor abnormalities that are unequivocal

**Supplemental Table 2: Behavioral testing schedule**

Age (Months)

| Tasks | 1 | 2 | 3 | 4 | 5 | 6 | 7 | 8 | 9 | 12 | 16 | 18 |
| --- | --- | --- | --- | --- | --- | --- | --- | --- | --- | --- | --- | --- |
| INAS | ✓ | ✓ |  |  |  |  |  |  |  |  |  |  |
| 1pair-OD |  |  |  | ✓ |  |  |  | ✓ |  |  |  |  |
| PD |  |  |  |  |  |  |  | ✓ |  |  |  |  |
| COD |  |  |  |  |  |  |  |  | ✓ |  |  |  |
| Detour-Reaching |  |  |  |  |  |  |  |  |  |  | ✓ |  |
| ODR |  |  |  |  |  |  |  |  |  | ✓ |  |  |
| VPC-Delay | ✓ |  |  | ✓ |  |  |  |  |  |  |  | ✓ |
| VPC-Spatial |  |  |  |  |  |  |  |  |  |  |  | ✓ |
| DNMS |  |  |  |  |  |  |  |  |  |  | ✓ |  |
| VS-OR |  |  |  |  |  |  |  |  |  |  | ✓ |  |

INAS: Infant Neurobehavioral Assessment Scale ^1^; 1pair-OD: One-pair object discrimination; PD: Pattern discrimination; COD: 24-hr concurrent discrimination ^2^; Detour-reaching task ^3^; ODR: Object Discrimination Reversal ^4^; VPC-Delay: Visual Paired Comparison with delays ^5^; VPC-Spatial: Visual Paired Comparison spatial versions ^6^; DNMS: trial-unique Delayed NonMatching-to-Sample ^7^; VS-OR: Visuospatial-Orientation Task ^8^.

Supplemental Table 3 - INAS behaviors and rating scale

| \|  \| **Behavior** \| **Description** \| **0** \| **Scores**  **1** \| **2** \| \| --- \| --- \| --- \| --- \| --- \| --- \| \|  \| Visual orientation \| Looking at a stationary stimulus presented in 4 different locations \| no contacts \| direct/ brief visual contact \| direct prolonged contact \| \| Orientation Responses \| Visual  following \| Following of a stimulus moving left to right \| contact  but no following \| starts to  follow but stops \| follows for the duration the stimulus moved \| \|  \| Attention  span \| Amount of attention on visual orientation and following \| lack of attention at all times \| attentive at least 25% of the time \| attentive at least 75% of the time \| \|  \| Auditory orientation \| Infant orients to a lip-smacking sounds made by an hidden experimenter \| no orientation \| partial head turning \| full head turn with visual inspection \| \|  \| Muscle tonus-prone \| Infant's ability to hold head up when placed on his belly \| flaccid tone/  head hangs down \| head lifted/  maintained < 3 s \| head lifted/ maintained > 3 s \| \| Neuromotor abilities \| Muscle tonus-supine \| Infant's ability to hold head up when placed on his back \| flaccid tone/ head hangs down \| head lifted/ maintained < 3 s \| head lifted/ maintained > 3 s \| \|  \| Pull to sit \| Infant's arm strength and head rising in response to being pulled from a supine to sitting position \| limbs extend and head lags \| arms moderately flex with no head lag \| resistance with attempts to turn over \| \|  \| Motor activity/  4 min spans \| Amount of movement \| motion for 1 minute \| motion for 2-3 minutes \| motion for  > 3 minutes \| \|  \| Locomotion/  4 min spans \| Amount of locomotion \| no locomotion \| weak attempts \| coordinated locomotion \| \| Motor  abilities \| Coordination/  4 min spans \| Quality of movements \| clumsy movements \| adequate movements \| agile movements \| \|  \| Response intensity \| Quality and quantity of vocal reactions \| mild intensity \| moderate intensity \| extremely loud \| \|  \| Fearfulness \| Fear grimaces or trembling \| none \| fear early in testing \| fear noted frequently \| \| Temperament measures \| Struggle  during testing \| Degree of squirming \| 25% of the time \| 50% of the time \| constant struggling \| \|  \| Irritability \| Amount of distress \| no apparent distress \| 50% of the time \| continuous distress \| \|  \| Consolability \| Ease of consoling infant during distress \| Impossible to console \| Difficult to console \| Easy to console \| |  |  |  |
| --- | --- | --- | --- | --- | --- | --- | --- | --- | --- | --- | --- | --- | --- | --- | --- | --- | --- | --- | --- | --- | --- | --- | --- | --- | --- | --- | --- | --- | --- | --- | --- | --- | --- | --- | --- | --- | --- | --- | --- | --- | --- | --- | --- | --- | --- | --- | --- | --- | --- | --- | --- | --- | --- | --- | --- | --- | --- | --- | --- | --- | --- | --- | --- | --- | --- | --- | --- | --- | --- | --- | --- | --- | --- | --- | --- | --- | --- | --- | --- | --- | --- | --- | --- | --- | --- | --- | --- | --- | --- | --- | --- | --- | --- | --- | --- | --- | --- | --- | --- |

**Supplemental Table 4**: **Definitions of behavioral measures in the Detour-Reaching Task**

| **Measures** | **Definitions** |
| --- | --- |
| Response initiation latency | Time from raising the screen at the start of the trial until the subject  makes contact with the test box or reward |
| Correct reaches | Retrieval of the reward on a trial |
| Success reaches | Retrieval of the reward on the first reach of the trial |
| Unsuccessful reaches | Reaching into the open side of the box but failing to retrieve the reward or dropping it |
| Barrier reaches | Reaching to the closed, transparent side of the box |
| Total reaches | Number of reaches on a trial |
| Perseverative reaches | A trial in which the first reach was made into the barrier and is repeated over and over. These reaches were not included in “Total reaches” |

**REFERENCES**

1. Schneider, M.L. & Suomi, S.J. Neurobehavioral assessment in rhesus monkey neonates (*Macaca mulatta*): developmental changes, behavioral stability, and early experience. *Infant behavior and development* **15**, 155-177 (1992).

2. Phillips, R.R., Malamut, B.L., Bachevalier, J. & Mishkin, M. Dissociation of the effects of inferior temporal and limbic lesions on object discrimination learning with 24-h intertrial intervals. *Behav Brain Res* **27**, 99-107 (1988).

3. Diamond, A. Rate of maturation of the hippocampus and the developmental progression of children's performance on the delayed non-matching to sample and visual paired comparison tasks. *Ann N Y Acad Sci* **608**, 394-426; discussion 426-333 (1990).

4. Meunier, M., Bachevalier, J. & Mishkin, M. Effects of orbital frontal and anterior cingulate lesions on object and spatial memory in rhesus monkeys. *Neuropsychologia* **35**, 999-1015 (1997).

5. Zeamer, A., Heuer, E. & Bachevalier, J. Developmental trajectory of object recognition memory in infant rhesus macaques with and without neonatal hippocampal lesions. *J Neurosci* **30**, 9157-9165 (2010).

6. Bachevalier, J. & Nemanic, S. Memory for spatial location and object-place associations are differently processed by the hippocampal formation, parahippocampal areas TH/TF and perirhinal cortex. *Hippocampus* **18**, 64-80 (2008).

7. Mishkin, M. & Delacour, J. An analysis of short-term visual memory in the monkey. *J Exp Psychol Anim Behav Process* **1**, 326-334 (1975).

8. Petrides, M. & Iversen, S.D. Restricted posterior parietal lesions in the rhesus monkey and performance on visuospatial tasks. *Brain Res* **161**, 63-77 (1979).
