# Supplementary material for: A two years longitudinal study of a transgenic Huntington disease monkey
Source: BMC Neurosci. 2014 Mar 3;15:36. doi: 10.1186/1471-2202-15-36 (PMC4015530; doi:10.1186/1471-2202-15-36)
Supplement: Additional file 2 — Supplemental Methods: Behavioral Testing. [file 1471-2202-15-36-S2.docx]

***Supplemental Methods: Behavioral Testing:***

***Measures of stimulus-reward associations***

To assess functioning of the striato-cortical loop, we used behavioral tasks for which performance is known to be affected by lesions of the striatum ^[1-3](#_ENREF_1)^. In addition, given that we needed to measure striatal-dependent functions across development, stimulus-reward association learning tasks used in each age increased in difficulty from the simplest 1-pair OD given at 4 and 8 months to the more challenging pattern discrimination (PD) given at 8 months and 20-pair concurrent discrimination (COD***)*** at 9 months of age. (Please see Supplemental Method for detailed procedures).

1. ***1-Pair Object Discrimination (1-pair-OD):*** A testing tray with three recessed food wells (2cm in diameter, 1cm deep and 13 cm apart from each other) was used for this task and two three-dimensional objects differing in color, shape and texture were selected. On the first trial, the two objects were positioned over the lateral wells of the test tray and both objects were baited. The object chosen by the monkey was designated the positive one for the remaining of the task. The pair was then presented for 30 trials per day with a noncorrection technique. For each trial, the left-right position of the positive object was pseudorandomly determined, and the trials were separated by 10-s intervals. The monkeys were trained until they reached a score of at least 28 correct out of 30 trials on one day (> 90%) followed the next day by a score of at least 24 correct out of 30 trials (80%).
2. ***Pattern Discrimination (PD):*** This task was administered in exactly the same way as the 1-pair-OD task, except that the two stimuli were two white plastic plaques (18 cm X 18 cm) instead of real objects. One plaque contained a black “**+**” sign in its center, whereas the other contained a black “🞏” in its center.
3. ***24-hr Concurrent Object Discrimination (24-hr COD):*** In this task, the two lateral food-wells of the testing tray were used. A series of 20 pairs of novel objects were selected, with one object in each pair designated positive (baited) and the other negative (unbaited). The two objects of the pair were presented simultaneously over the lateral well of the test tray, and the monkey was allowed to displace one of them. After a 20-s intertrial interval, the next pair of objects was presented for choice in the same manner, and so on until all 20 pairs of objects had been presented once each. Twenty-four hours later the same 20 pairs of objects were presented again, in the same order as before, and so on day after day. The left-right positions of the positive and negative objects of each pair were changed pseudorandomly each day. Testing continued until the animal attained a criterion of 90 correct responses in 100 consecutive trials (five daily sessions). A noncorrection procedure was used throughout.

***Measure of flexible behavioral and cognitive inhibition and impulsivity***

To assess the functioning of the frontal cortex, we use two sensitive tasks: object discrimination reversal (ODR) and Detour-reaching/barrier task at 12 months and 16 months, respectively. (Please see Supplemental Method for detailed procedures and Supplemental Table 4).

1. ***Object Discrimination Reversal Task:*** In this task, two novel objects formed a single discrimination problem. Animals had first to learn which of the two objects was associated with the food reward (Acquisition phase) followed by 6 reversals. During the first trial of the acquisition phase, both objects covered a food reward and the object selected by the animal became the rewarded object (S^+^) for the remaining trials of the phase. Left/right positions of the S^+^ varied according to a pseudorandom sequence. Animals were given a total of 30 trials per day at 5-sec intertrial intervals until they reached a criterion of 28 correct choices over 30 trials (> 90%) on one day followed by a criterion of 24 correct choices in 30 trials (> 80%) on the next day. Upon reaching this criterion, the reward contingency was switched so that the S^+^ became S^-^ and vice versa. The animal was again given 30 trials per day until the same criterion was met, after which the reward contingency was switched again. The animals were given a total of six reversals. During acquisition phase and reversals, incorrect choices were corrected by re-running the erroneous trial with the S^+^ covering the reward and the S^-^ placed beside the empty well. This correction was repeated as many times as necessary until the animals displaced the S^+^.
2. ***Detour-reaching/barrier task:*** The apparatus consists of a small transparent box (5” x 5” x 4”), open on only one side and fixed on a tray. The box could be located at different positions on the tray and could be rotated so that its open side could be changed. The position of the reward within the box (entrance but out of box, one third in, half way in, and three quarter in), of the box on the tray (left, right, center), and orientation of the open side of the box relative to the subject (front, left, right) were parametrically varied. Subjects were allowed unlimited time to retrieve the reward as long as they continued attempting to respond. A maximum of 3 min during which no responses occurred terminated the trial and was scored as a “failure”. Training consisted of 7 testing days as follows. Day 1**:** Fifteen trials were given with the opening of the box facing towards the animal, such that all trials involve a direct reach for the reward not requiring the animal to inhibit reaching at the barrier. Days 2-4: Test sessions consisted of 18 trials, in which the trials required the animal to either make a direct reach (box opening facing the animal) or to negotiate a detour (box opening facing right or left of the animal). These two trial types were intermixed within a session. Days 5-7: The plastic box was positioned at the center of the box for all trials. Daily test sessions consisted of 21 trials requiring the animal to negotiate a detour on one side five times in the row followed by a switch requiring the animal to negotiate a detour in the other side five time in the row and so on (e.g., box opening on the left, left, left, left, left then box opening on the right, right, right, right, right, etc…). These trials were included to increase the tendency for animals to exhibit perseverative responses, defined as the successive repetitions of a previously rewarded reach. All testing was video-recorded and the video-tapes were subsequently analyzed by an experimenter blind regarding the animal treatment. Several measures of performance were included and are listed in Supplemental Table 4.

***Measures of object and spatial recognition memory functions:***

Sensitive measures of medial temporal lobe and hippocampal functions were assessed with two recognition memory tasks: VPC tasks (1, 4, and 8 months) and DNMS (16 months). (Please see Supplemental Method for detailed procedures).

1. ***VPC tasks:*** For all VPC tasks, the apparatus consisted of a 19” TV monitor positioned on a table at the animal’s eye level and a video camera (Sony Digital8 TRV-140) mounted above the screen and positioned to capture the monkey’s eye movements. The camera output was fed into a time/date generator connected to a VCR (JVC HR-S4800U) that recorded eye movements for each trial and into a TV monitor to monitor the animal’s looking behavior during the task. Stimuli were pictures of variegated objects varying in shape and texture (trees, animals, tools, etc…) and presented in their colored version (color pictures) or in their black/white version (BW pictures). These stimuli were selected from a pool of 800,000 clipart images (Nova Art Explosion 800,000 Clip Art). Novel pictures were selected for each trial and were never repeated from tasks to tasks. All images were delivered to the monitor via a computer controlled by the experimenter
2. ***Delayed Nonmatching-to-Sample (DNMS) (16 months):*** The stimuli were drawn from a pool of 1000 objects varied in size, shape and color, which were novel to the animal prior to testing. On each trial, the monkey was presented with a single object overlying the center well containing a food reward. After displacing the object and retrieving the reward, a 10-s delay was imposed and followed by a choice test in which the familiar object and a novel baited object each covering the lateral wells were presented for choice. New objects were used for each trial and 20 trials, separated by a 30-s inter-trial interval, were given per daily session until the monkey met the criterion of 90 correct responses in 100 consecutive trials. After achieving the learning criterion, their recognition memory was taxed further with performance tests including 100 trials at delays of 30, 60 and 120s, and 50 trials at delay of 600s.

.

**Pre-Training:** At 1.5 and 4 months of age, the infant was held by an experimenter approximately 40 cm in front of the TV monitor. At 18 months of age, the monkey was seated in a custom made plexiglass primate chair (Crist Instruments, Hagerstown, MD) that fit the animal’s size. For 3 days prior to the start of formal testing, the 1.5-month-old infants were brought into the testing room where they watched a Disney cartoon for an increasing period of time each day (15, 20 and 25 minutes). A 20-min pre-training session was given for only one day when the animals reached 4 months of age. At 18 months of age, the animals were first trained over a period of a few days to enter and acclimate to the primate chair, and were then brought into the testing apparatus for one day to view a Disney cartoon. The animals were neither food deprived nor water restricted and remained in the testing apparatus for no more than 45 minutes a day.

**VPC task with delays:** All monkeys were tested on the visual paired-comparison task (VPC) at 1, 4, and 16 months. Each VPC trial was divided into a familiarization phase and a retention phase. In the familiarization phase, a stimulus appeared and remained in the center of the screen until the monkey spent a cumulative 30 seconds looking at the sample image as estimated on-line by the experimenter. After a delay, which varied from 10, 30, 60 and 120 seconds and was intermixed across each testing session, two 5-s retention tests separated by a 5-s delay were given. During these two retention tests, the sample picture and a novel picture were displayed side-by-side 12 cm apart on the screen for 5 seconds after the animal initially looked at one of the two pictures. The left/right position of the two pictures was reversed during the two retention tests, and on the first retention test, the left/right position of the novel picture varied pseudo-randomly. The screen remained black during all delay periods, and at each age, ten trials were given at each delay at 30-s inter-trial interval (ITI). Animals were first tested with pictures of color objects and then with pictures of BW objects. The animals were given 6 – 10 trials per day and did not receive food treats during the testing period at 1 and 4 months of age, but were given mini-M & M’s during random ITIs at 18 months to encourage them to remain focused on the screen.

**VPC-Spatial tasks:** Three versions of the VPC paradigm (VPC-Location, VPC-Object-in-Place and VPC-Control) were used to measure spatial memory as described by Bachevalier and Nemanic (2008) ^[4](#_ENREF_4)^ at 18 months of age.

***VPC-Spatial Location*:** In this version of the VPC task, the measure of preferential viewing is to a specific location on the screen for each trial, and thus, the stimuli used for the familiarization phase and the retention tests were identical but appeared on different locations on the screen. In the familiarization phase, a single image appeared anywhere on the screen. In the retention phase, the familiar image appeared on the same location and an identical image appeared in a different location of the screen. An algorithm in the VPC program randomly selected the locations of the stimuli for each trial, with the convention that the images could not overlap and that at least 2–10 cm separated the two images in any direction. Finally, only one retention test of 8 seconds duration was used for this task.

***VPC-Object-in-Place*:**  In this version of the task, the stimulus consisted of a group of 5 objects (spaced 2 cm apart) presented as an array over a white rectangular background. For each trial, the 5 objects used for the retention tests were identical to those used for the familiarization phase, but in the new image the location of three objects was re-arranged. Thus, the only difference between familiar and novel stimuli was the location of the five objects relative to each other. Extreme care was taken to ensure that the re-arranged objects occupied the exact same position (identical x and y coordinates) as the previous objects.

***VPC-Control task*:** This control VPC task was designed to ascertain that any lack of novelty preference in the VPC-Object-in-Place task was not a reflection of a difficulty in scanning the multiple objects on the images. Thus, as for the VPC-Object-in-Place task, the images consisted of five objects (spaced 2 cm apart) grouped together on a white rectangular background. For each trial, the comparisons in the retention tests was between the image seen in the familiarization phase and a new image consisting of two of the five objects seen in the familiar image and three new objects. The new objects could occupy the position of any of the five objects shown in the familiar image. Again, the new object was positioned at the precise “x” and “y” coordinates of the center of the object that it had replaced and was selected to match the color of the other objects on the image to reduce to a minimum a pop-out effect.

**Data Analysis for VPC tasks:** For each trial, the video recordings of the retention tests were analyzed frame-by-frame to calculate the amount of time the animal spent looking towards the familiar and the novel pictures (see for details Pascalis and Bachevalier, 1999 ^[5](#_ENREF_5)^). Two observers, each blind to the position of the novel image and to the lesion group, scored the videotapes independently (inter-observer reliability: pearson *r* = 0.925). The length of time it took the animal to familiarize with the first picture (Total Familiarization Phase) and total time looking at both pictures during the retention tests (Total Retention Time) were recorded and analyzed. The percent time looking to the novel picture across the two retention tests (Percent Novelty) was also calculated [(time looking at novel / total retention time) X 100)]. Trials for which the total looking time during the two retention tests was less than one second were rejected. Only one or two trials per animals met this criterion.

***Measures of visuospatial abilities***

To assess the contributions of the frontal-striatal system to fine motor control and associative learning, we use VS-OR (or lifesaver task) at 16 months of age. (Please see Supplemental Method for detailed procedures).

***Visuospatial orientation (VS-OR):*** In this task, the monkey was required to free a ring-shaped candy (“Lifesaver”) by moving it along metal rods bent into detour patterns or routes of varying complexity at 16 months of age. A testing tray with a vise at its center onto which metal-rods were secured was used. Metal-rods were either straight for the pre-training phase or with 1-3 bends for the “Easy routes” and 4-5 bends for the “Difficult routes” (see Figure 1 in Bachevalier et al., 1991 ^[6](#_ENREF_6)^ for details of the metal-rods used). On each trial the central pole of a metal-rod route was inserted into the vise and a lifesaver was threaded along the rod back to the starting point, located on either the right or the left side of the central pole. Monkeys were required to retrieve the Lifesaver by threading it to the free end of the rod. Testing was divided into three stages. The pretraining stage consisted of 12 daily sessions in which the animal learned to remove the Lifesaver from a straight rod place in one of four different orientations (up, right, left and toward the monkey). For each orientation, the monkey was given six practice trials in which the experimenter helped the animal free the Lifesaver if necessary. These practice trials were followed by six test trials at each orientation on each of three days. The monkey performed these test trials on its own, and the latency to retrieve the Lifesaver was recorded. In the second stage, twelve “easy” routes were presented pseudorandomly three times each for a total of 36 trials. The animals received six of these trials per day for six consecutive days, and a maximum of 45 seconds was allotted to solve each route (e.g. retrieve the reward). Finally, in the third stage, twelve “difficult” routes were presented in the same manner as in the second stage, and a maximum of 120 seconds was allowed to solve each one.

**REFERENCES**

1. Buerger AA, Gross CG, Rocha-Miranda CE. Effects of ventral putamen lesions on discrimination learning by monkeys. J Comp Physiol Psychol 1974; 86:440-6.

2. Divac I, Rosvold HE, Szwarcbart MK. Behavioral effects of selective ablation of the caudate nucleus. J Comp Physiol Psychol 1967; 63:184-90.

3. Fernandez-Ruiz J, Wang J, Aigner TG, Mishkin M. Visual habit formation in monkeys with neurotoxic lesions of the ventrocaudal neostriatum. Proc Natl Acad Sci U S A 2001; 98:4196-201.

4. Bachevalier J, Nemanic S. Memory for spatial location and object-place associations are differently processed by the hippocampal formation, parahippocampal areas TH/TF and perirhinal cortex. Hippocampus 2008; 18:64-80.

5. Pascalis O, Bachevalier J. Neonatal aspiration lesions of the hippocampal formation impair visual recognition memory when assessed by paired-comparison task but not by delayed nonmatching-to-sample task. Hippocampus 1999; 9:609-16.

6. Bachevalier J, Landis LS, Walker LC, Brickson M, Mishkin M, Price DL, Cork LC. Aged monkeys exhibit behavioral deficits indicative of widespread cerebral dysfunction. Neurobiol Aging 1991; 12:99-111.
